# Developing a flexible, high‐efficiency *Agrobacterium*‐mediated sorghum transformation system with broad application

**DOI:** 10.1111/pbi.12879

**Published:** 2018-02-06

**Authors:** Ping Che, Ajith Anand, Emily Wu, Jeffry D. Sander, Marissa K. Simon, Weiwei Zhu, Amy L. Sigmund, Gina Zastrow‐Hayes, Michael Miller, Donglong Liu, Shai J. Lawit, Zuo‐Yu Zhao, Marc C. Albertsen, Todd J. Jones

**Affiliations:** ^1^ DuPont Pioneer Johnston IA USA; ^2^ Present address: 1969 W. Grand Canyon Dr Chandler AZ 85248 USA

**Keywords:** sorghum transformation, Africa sorghum varieties, *Agrobacterium*, genome modification, ternary vector, CRISPR/Cas9

## Abstract

Sorghum is the fifth most widely planted cereal crop in the world and is commonly cultivated in arid and semi‐arid regions such as Africa. Despite its importance as a food source, sorghum genetic improvement through transgenic approaches has been limited because of an inefficient transformation system. Here, we report a ternary vector (also known as cohabitating vector) system using a recently described pVIR accessory plasmid that facilitates efficient *Agrobacterium*‐mediated transformation of sorghum. We report regeneration frequencies ranging from 6% to 29% in Tx430 using different selectable markers and single copy, backbone free ‘quality events’ ranging from 45% to 66% of the total events produced. Furthermore, we successfully applied this ternary system to develop transformation protocols for popular but recalcitrant African varieties including *Macia*,* Malisor 84‐7* and *Tegemeo*. In addition, we report the use of this technology to develop the first stable CRISPR/Cas9‐mediated gene knockouts in Tx430.

**Abbreviations**
CIV: co‐integrating vector
Vir: virulence
PMI: phosphomannose isomerase
Phi: phosphite
Pi: phosphate
YFP: fluorescent protein
CENH3: centromere‐specific histone H3
AGMT: *Agrobacterium*‐mediated transformation
PTXD: phosphonate dehydrogenase
NPTII: neomycin phosphotransferase II
CRISPR: clustered regularly interspaced short palindromic repeats
Cas: CRISPR‐associated
PAM: protospacer adjacent motif

## Introduction

Tolerant to hot and dry conditions, sorghum (*Sorghum bicolor* L.) is the fifth most widely planted cereal crop in the world and the second most important cereal (after maize) in sub‐Saharan Africa (Awika and Rooney, 2004; Hiei *et al*., 2014; Ji *et al*., 2013; Liu and Godwin, 2012). It is estimated that about 500 million people worldwide rely on sorghum as their daily staple food source (Awika and Rooney, 2004; Che *et al*., 2016; Hiei *et al*., 2014; Liu and Godwin, 2012). However, sorghum grain is seriously micronutrient deficient in pro‐vitamin A (β‐carotene), in the bioavailability of iron and zinc, and is poor in protein digestibility (Che *et al*., 2016). Due to the limitation of traditional breeding, plant biotechnology has been demonstrated as an essential component for crop biofortification and improving crop agronomic performance. However, the lack of an efficient sorghum transformation system for genetic modification, especially for the important African varieties, has impeded sorghum research aimed at elucidating mechanisms for heat and drought resistance, increasing yield and improving grain quality.

In principle, a successful transformation system relies on a combination of factors including a selectable marker, efficient gene delivery and responsive tissue culture. Although significant progress has been made in recent years and a transformation efficiency of ~21% can be achieved with microprojectile bombardment (Liu and Godwin, 2012), the event quality from particle bombardment‐derived plants is typically low and rarely results in precise single‐copy insertions of only the desired DNA sequence or ‘quality events’ (QE) (see Experimental Procedures for ‘QE’ definition). While *Agrobacterium*‐mediated transformation (AGMT) is more amenable to low and single‐copy insertions of precise DNA sequence, optimized tissue culture protocols are limited to Tx430 and transformation efficiencies of total event production are only 10% (Wu *et al*., 2014). More virulent *Agrobacterium* strains such as AGL1 have achieved higher transformation frequencies; however, the event quality decreased even more significantly, resulting in fewer overall usable quality events (Wu *et al*., 2014; Zhi *et al*., 2015). When compared with transformation methods for other cereal crops, such as corn and rice, sorghum transformation lags far behind (Ji *et al*., 2013).

**Figure 1:**
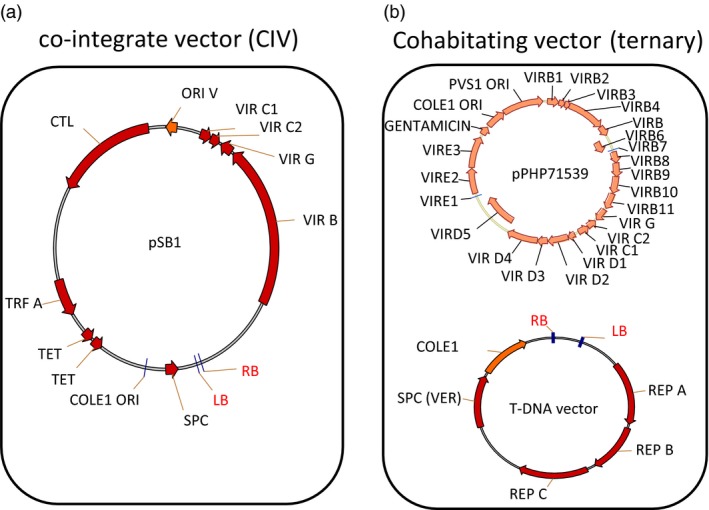
Schematic representation of the molecular components of (a) CIV and (b) ternary vector systems. pPHP71539 works as the accessory plasmid for the ternary vector system.

Binary vectors are ideal for introducing genes into crop plants because of their ability to integrate transgenes in low and single copy. The broader application of AGMT has been achieved by implementing so‐called super‐binary, co‐integrate vectors (CIV) resulting in vastly improved crop transformation, especially for cereals (Ishida *et al*., 1996). However, the super‐binary plasmid pSB1 (Figure 1a) has limited number of *vir* genes (B, G and part of C) included on the plasmid and requires a co‐integration step to generate the final construct. In addition, the large plasmid size also presents a challenge for high‐throughput vector construction (Ishida *et al*., 1996). To address these issues and improve plant transformation, Anand *et al*. (2017a) designed a series of pVIR vectors [pPHP70298, pPHP71539 (Figure 1b) and pPHP79761] containing an optimal set of *vir* genes. These genes were assembled into ternary vector (also known as cohabitating vector) (Anand *et al*., 2017a,2017b) in which a T‐DNA‐less accessory pVIR plasmid and a T‐DNA transfer competent binary plasmid are cotransformed into an *Agrobacterium* cell and used for plant transformation (Figure 1b). These pVIR vectors have many desirable features, including smaller vector sizes, enhanced vector stability and amended *vir* genes for enhanced T‐DNA gene delivery. Furthermore, the ternary vector system was demonstrated to improve maize transformation (Anand *et al*., 2017b). This encouraged us to assess it for enhancing sorghum transformation in combination with the media optimization previously established (Wu *et al*., 2014).

Using surfactants and the ternary vector system, we developed a highly efficient and broadly applicable sorghum transformation system that works with a range of selectable markers for Tx430 as well as the recalcitrant Africa varieties including *Macia*,* Malisor 84‐7* and *Tegemeo*. We applied our sorghum transformation system to generate high‐quality transgenic events and demonstrated its potential to support efficient genome editing by creating the first stable knockouts in sorghum using CRISPR/Cas gene editing technology.

## Results

### Enhancing *Agrobacterium*‐mediated sorghum transformation frequency with surfactant

**Figure 2:**
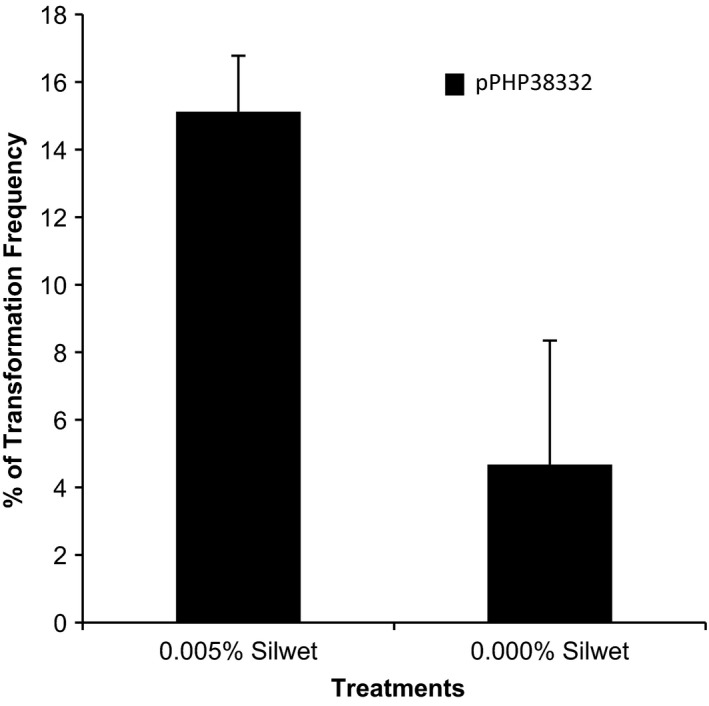
Effect of surfactant Silwet‐70 on transformation frequency in Tx430. Error bars indicate the ±SD from three treatments with more than 100 embryos per treatment.

We chose to investigate whether the surfactant Silwet‐70 could enhance sorghum transformation as has been demonstrated transiently for both corn and wheat transformation (Cao *et al*., 2014; Wu *et al*., 2003). We tested the effects of Silwet‐70 application on *Agrobacterium*‐mediated immature embryo transformation of Tx430 using the transformation protocol published by Wu *et al*. (2014). Plasmid pPHP38332 (Figure S1a) was constructed as a CIV vector from super‐binary vector pSB1 (Figure 1a) and transferred into *Agrobacterium* auxotrophic strain LBA4404 Thy‐ (thymidine mutant), a strain for better control bacterial overgrowth during tissue culture compared to strain LBA4404 previously used (Wu *et al*., 2014). The plasmid pPHP38332 carries a 4.6 kb T‐DNA harbouring the phosphomannose isomerase (*PMI*) selectable marker gene and a yellow fluorescent protein (YFP) visual reporter gene. As shown in Figure 2, the application of 0.005% Silwet‐70 significantly increased the overall transformation frequency about threefold and no deleterious effects were observed (data not shown). These data are consistent with the use of Silwet to increase *Agrobacterium*‐mediated gene delivery in corn and wheat (Cao *et al*., 2014; Wu *et al*., 2003). Based on these results, 0.005% Silwet‐70 was applied to all subsequent experiments throughout this study.

### Enhancing *Agrobacterium*‐mediated gene delivery using cohabitating vectors

**Figure 3:**
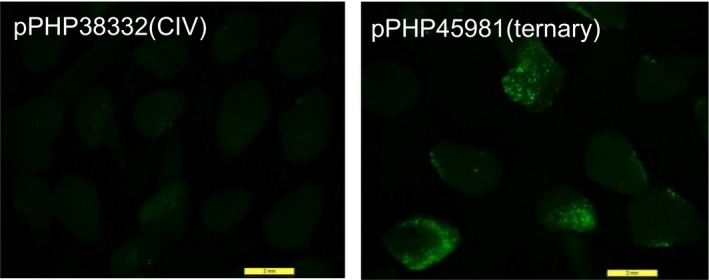
T‐DNA delivery determined by YFP transient assay. Sorghum Tx430 immature embryos were infected with *Agrobacterium* carrying either pPHP38331(CIV) or pPHP45981(ternary) with pPHP71539 as accessory plasmid. T‐DNA delivery was represented by the number of transgenic cells exhibiting YFP fluorescence and the fluorescence intensity on the surface of sorghum embryos.

To evaluate T‐DNA delivery using the ternary system in sorghum, we transformed binary vector pPHP45981 (Figure S1b) containing the T‐DNA (7.7 kb) with the *PMI* selectable marker and *YFP* into the *Agrobacterium* strain LBA4404 Thy‐ harbouring the pVIR accessory plasmid pPHP71539. The analogous CIV construct, pPHP38332 (Figure S1a), was transformed into the same *Agrobacterium* strain LBA4404 Thy‐ minus pPHP71539 plasmid. The transient gene delivery for each system was assessed by visually evaluating the number of fluorescent expressing foci on the surface of sorghum embryos after 3 days of *Agrobacterium* infection on cocultivation medium. As shown in Figure 3, the embryos infected with pPHP45981(ternary) showed both a greater number of infected cells and substantially enhanced fluorescence intensity compared to embryos infected with pPHP38332(CIV). This observation corroborated the maize results demonstrating superior T‐DNA delivery with the ternary construct, compared to the CIV construct (Anand *et al*., 2017b), a preferred design for sorghum transformation. The ternary design also offers simple and versatile vector assembly requiring no co‐integration step, which can occasionally result in vector integrity, complicating vector assembly and mobilization into multiple *Agrobacterium* strains.

Having confirmed that the ternary vector enhanced transient T‐DNA delivery in sorghum, we investigated whether this translated into improved stable transformation in sorghum. To this end, we designed two ternary donor constructs derived from pPHP82637 (Figure S1e), pPHP78152 and pPHP78233 with T‐DNA sizes of 17–18 kb, respectively. The T‐DNA harbours a *PMI* selectable marker and three different proprietary genes for pPHP78152 and four for pPHP78233. The plasmids were mobilized in strain LBA4404 Thy‐ harbouring the accessory plasmid pPHP71539 and were used to transform Tx430 embryos. T0 transformation efficiencies for the two ternary constructs were 29% and 25%, respectively (Table 2). The stable transformation frequency observed with the larger size T‐DNA (17–18 kb) in the ternary vectors was considerably higher compared to transformation frequency (15%) observed with the CIV construct pPHP38332 (T‐DNA, 8 kb) (Table 1).

**Table 1:** Transformation efficiency and event quality using CIV transformation system and *PMI* as selectable marker

Construct	Variety	Transformation efficiency %	Quality events %
pPHP38332 (4.6 kb T‐DNA)	*Malisor 84‐7*	9.3 ± 2.8	61%
	*Tegemeo*	6.4 ± 2.7	NA
	*Macia*	0.0 ± 0.0	NA
	Tx430	15.1 ± 1.7	66%

Data were presented as the average ± SD of three biological replications.

Thirty embryos were used for each biological replication. NA: Not available.

Next, we evaluated the event quality of the transgenic plants produced with the ternary vector system. Higher transformation frequencies can often be accompanied by an undesirable increase in copy number of T‐DNA insertions and backbone integration (Oltmanns *et al*., 2010; Zhi *et al*., 2015). Multicopy events have the potential to affect transgene expression and stability as well as impede regulatory assessment (Oltmanns *et al*., 2010; Zhi *et al*., 2015).

To assess event quality, T0 plants were generated using pPHP38332(CIV), pPHP78152(ternary) or pPHP78233(ternary) as detailed in Experimental Procedures. T0 plants were subjected to detailed molecular event characterization as previously reported (Wu *et al*., 2014; Zhi *et al*., 2015). As represented in Table 2, 54% and 65% of the events produced with the ternary constructs were determined to be single‐copy insert, backbone‐minus quality events, which was comparable to quality event frequency determined for the CIV construct pPHP38332 (66%) with a relatively smaller T‐DNA (Table 1). Based on the above observation, it was concluded that the ternary vector results in a higher transformation efficiency with minimal to no impact on the quality of events produced in sorghum. In practical terms, this means fewer immature embryos are required to produce the same number of quality events compared to the CIV transformation system.

**Table 2:** Transformation efficiency and event quality using ternary transformation system and *PMI* as selectable marker

Construct	Variety	# of embryos	# of events	Transformation efficiency %	Quality events %
pPHP78152 (17 kb T‐DNA)	*Malisor 84‐7*	561	53	9.4%	47%
	*Tegemeo*	420	8	1.9%	NA
	*Macia*	440	3	0.7%	NA
	Tx430	350	103	29%	54%
pPHP78233 (18 kb T‐DNA)	*Malisor 84‐7*	599	33	5.5%	51%
	*Tegemeo*	450	5	1.1%	NA
	*Macia*	521	7	1.3%	NA
	Tx430	400	99	25%	65%

NA, Not available.

### Transformation for different sorghum varieties

Genotype dependence is a major limitation of an immature embryo‐based transformation system currently observed in the cereals, corn, rice, wheat and sorghum (Harwood, 2012; Hiei *et al*., 2014; Que *et al*., 2014). Although sorghum transformation has been improved for several model varieties in the past decade (Ji *et al*., 2013), no transformation has ever been carried out in the background of native African varieties. To broaden the application of the sorghum transformation technology and to facilitate sorghum research for yield and quality improvement in African countries, we further evaluated transformability of three popular Africa varieties, *Macia*,* Malisor 84‐7* and *Tegemeo* using the ternary vector design. We successfully transformed both *Malisor 84‐7* and *Tegemeo* using the CIV construct pPHP38332 with transformation frequencies of 9.3% and 6.4%, respectively (Table 1). However, with the CIV vector, *Macia* was found to be highly recalcitrant to transformation and no events were generated from 5000 embryos infected under the same culture conditions (Table 1). In contrast, using the ternary vector design with the accessory plasmid pPHP71539 and binary vectors pPHP71852 and pPHP78233, we demonstrated successful transformation of all three African varieties (Table 2). The variety *Tegemeo* exhibited a lower transformation frequency with the ternary vector compared to the CIV construct (Tables 1 and 2) which we speculate could be due to the larger size of the T‐DNA in the ternary (17 and 18 kb) vs the CIV vector (4.6 kb). Nonetheless, these results strengthen our finding that enhanced T‐DNA delivery using the accessory plasmid pPHP71539 can broaden the transformability for various sorghum varieties. This to our knowledge is the first successful demonstration of AGMT in African sorghum varieties.

### Transformation using alternative selection markers

To broaden the applications of the ternary vector for transformation technology and provide extra flexibility for academic and industrial application, we tested alternate selectable markers for sorghum transformation. Two different selectable markers neomycin phosphotransferase II (*NPTII*)/G418 and a newly described Phosphonate dehydrogenase (*PTXD*)/phosphite (Phi) were evaluated for sorghum transformation.

*NPTII* is a widely used selectable marker gene that has been utilized successfully in corn transformation and previously described for sorghum transformation using particle bombardment (Ji *et al*., 2013; Liu and Godwin, 2012). Using the ternary construct with a T‐DNA vector (pPHP81561, Figure S1c) containing the *NPTII* gene and following a transformation procedure described by Wu *et al*. (2014), we optimized the concentration of G418 at 250 mg/L and achieved a transformation efficiency as high as 26% in Tx430 (Table 3), of which about 45% of the events were quality events. This is comparable to the *PMI* selectable marker system in terms of transformation (25%–29%) and quality event production efficiencies (54%–65%) (Table 2).

**Table 3:** Transformation efficiency and event quality of five independent experiments using ternary transformation system and *NPTII* as selectable marker

Construct	Experiment	# of embryos	# of callus	# of callus with shoots	Callus %	Transformation efficiency %	Quality events %
pPHP81561	1	209	62	44	30%	21%	45%
	2	115	30	25	26%	22%	
	3	115	29	24	25%	21%	
	4	121	40	32	33%	26%	
	5	117	46	29	39%	25%	

Given the concerns of using antibiotic and herbicide genes as selectable markers (Miki and McHugh, 2004), we further explored the possibility of using the bacterial *PTXD* gene as a positive selectable marker for *Agrobacterium*‐mediated sorghum transformation. Phosphite (Phi) has a similar molecular structure as phosphate (Pi). Unlike Pi, however, which can be used directly by plant cells as bioavailable phosphorus, Phi cannot be metabolized and plants that are grown on phosphite‐containing media are starved of phosphorus. It is reported that PTXD catalyses the conversion of Phi into inorganic Pi that can then be used by plant cells as a phosphorus source (Lopez‐Arredondo and Herrera‐Estrella, 2013). This enables transgenic plant cells carrying the *PTXD* gene to survive on the medium contain Phi as the sole phosphorus source. This selection system (*PTXD*/Phi) has been reported in corn, tobacco and Arabidopsis transformation (Lopez‐Arredondo and Herrera‐Estrella, 2013; Nahampun *et al*., 2016).

**Figure 4:**
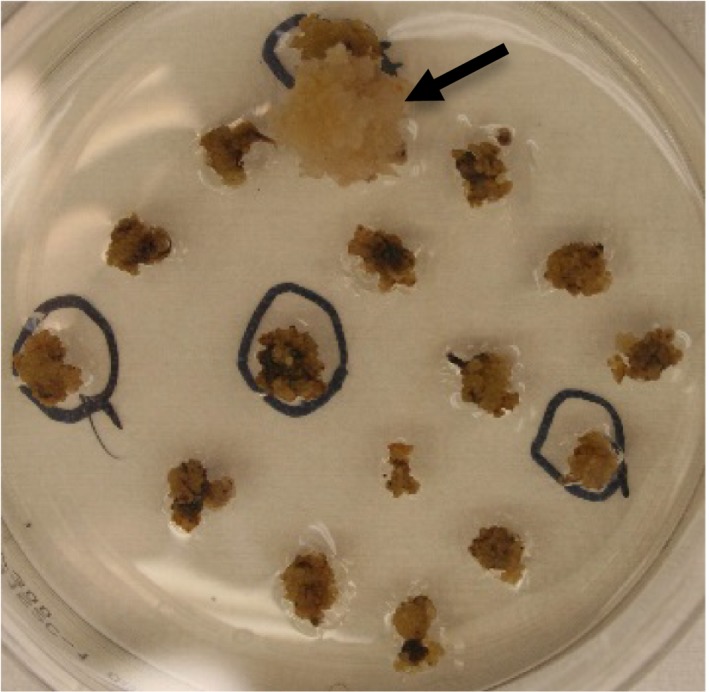
Stringent *PTXD*/Phi selectable system. Picture represents the development of 6 weeks old transgenic and nontransgenic callus on callus induction medium containing 300 mg/L Phi_._ The transgenic callus is indicated by the arrow.

The standard *Agrobacterium*‐mediated sorghum transformation procedure consists of six sequential steps: *Agrobacterium* infection, cocultivation, resting, callus development with selection, shoot and root induction (Wu *et al*., 2014). To develop the *PTXD*/Phi selectable system for sorghum transformation, we constructed pPHP70444 (Figure S1d) harbouring the *PTXD* gene, driven by a maize ubiquitin promoter and intron, in a ternary vector and tested for transformation using the modified transformation procedure either with or without a ‘resting’ step after cocultivation. We found that by imposing *PTXD*/Phi selection immediately and bypassing the resting step after cocultivation provided more stringent selection and supported robust callus development (Figure 4). This resulted in transformation efficiency up to 6% without detectable escapes and an average quality event frequency of 47% (Table 4).

**Table 4:** Transformation efficiency and event quality of four independent experiments using ternary transformation system and *PTXD* as selectable marker

Construct	Experiment	# of embryos	# of event with shoots	Transformation efficiency %	Quality events %
pPHP70444	1	100	4	4%	47%
	2	100	3	3%	
	3	100	4	4%	
	4	100	6	6%	

### Targeted sorghum genome editing using CRISPR/Cas9 system

**Figure 5:**
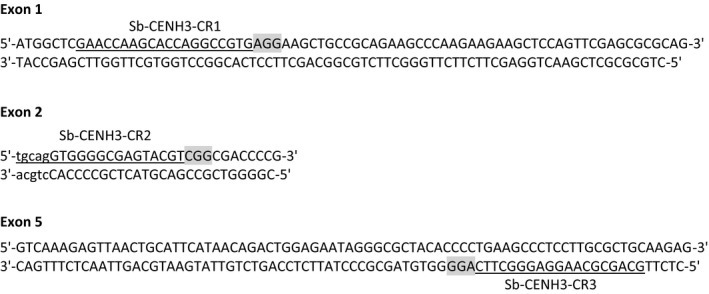
Diagram of the DNA sequence of the target region of *Sb‐CENH3* gene. The three‐nucleotide sequences highlighted in grey indicate the corresponding PAM motifs recognized by the Cas9 protein. crRNA hybridization targets for three different gRNAs are underlined. Exons and introns are represented by the uppercase and lowercase letters, respectively.

Targeted genome editing using the CRISPR/Cas system has proven to be a powerful tool for crop engineering and has been successfully applied to maize, rice and numerous other plant species to generate stable genome edited events with targeted modifications (*e.g*. insertions, deletions and replacements) (Bortesi and Fischer, 2015; Jiang *et al*., 2013; Svitashev *et al*., 2015). In sorghum, targeted insertions and deletions induced by CRISPR/Cas system have also been observed transiently two weeks after *Agrobacterium*‐mediated transformation of immature sorghum embryos (Jiang *et al*., 2013). To assess CRISPR/Cas for generating stable gene knockouts in sorghum using the ternary vector system, we chose to knock out the sorghum centromere‐specific histone H3 (*Sb‐CENH3*) because of its important function in the regulation of chromosome segregation and the potential to utilize *Sb‐CENH3* knockouts in the development of a sorghum haploid inducer (Ravi and Chan, 2010). *Sb‐CENH3* in sorghum was identified based on sequence similarity to *At‐CENH3* (Ravi and Chan, 2010) and subsequently cloned and sequenced (Figure S2). Cas9 target sequences were selected by identifying all NGG PAMs recognized by *Streptococcus pyogenes* Cas9 (spCas9) and their preceding 20 nucleotides in the *Sb‐CENH3* gene. Three targets sites were selected based on their location and orthogonality of cognate gRNAs engineered using guanine to pair with the 20th and most distal nucleotide to facilitate expression from the U6 promoter (Figure 5).

Fewer than 300 sorghum immature embryos were utilized for AGMT for each ternary construct and subsequent tissue culture to generate stable genome edited events using G418 selection. The overall regeneration efficiency ranged from 8% to 16% for the three constructs targeted to the essential *Sb‐CENH3* gene (Table 5) which is much lower than that using pPHP81561 (Table 3) for conventional transformation and the same selectable marker system. We expect the lower efficiency could be due to potential lethality of biallelic *Sb‐CENH3* knockouts (see footnote for the definition in Table 6) as has been observed in maize and *Arabidopsis* (Kelliher *et al*., 2016; Ravi and Chan, 2010). All the T0 plants were analysed by deep sequencing to identify mutations at the target sites (see Experimental Procedures for detail). As shown in Table 6 and Table S2, the targeted editing efficiency (see footnote for the definition in Table 6) was 37%–40% based on the transgenic plants analysed and targeted knockout efficiency (see footnote for the definition in Table 6) was in the range of 20%–37%. No biallelic knockouts were ever identified for all three constructs. This is consistent with a hypothesis of lethality for biallelic *Sb‐CENH3* mutations. Based on the information described above, the knockout efficiency per embryo transformed is about 1%–5% (Table 6 and Table S2). We expect that the overall knockout efficiency would be higher if editing were conducted with genes that were nonlethal as biallelic mutations.

**Table 5:** Regeneration efficiency for CRISPR/Cas9‐mediated *Sb‐CENH3* gene editing using ternary transformation system

gRNA	# of embryos	# of regenerated T0 plants	Regeneration %
Sb‐CENH3‐CR1	298	39	13%
Sb‐CENH3‐CR2	285	23	8%
Sb‐CENH3‐CR3	257	42	16%

**Table 6:** CRISPR/Cas9‐mediated gene editing efficiency for *Sb‐CENH3*

gRNA	# T0 plants analysed^a^	# T0 plants with edit^b^ (biallelic)	% T0 plants with edit (biallelic)	# T0 plants with a knockout^c^ (biallelic)	% T0 plants with a knockout^c^ (biallelic)	Knockout frequency per embryo transformed^d^
Sb‐CENH3‐CR1	31	12 (5)	39% (16%)	9 (0)	29% (0%)	3%
Sb‐CENH3‐CR2	15	6 (5)	40% (33%)	3 (0)	20% (0%)	1%
Sb‐CENH3‐CR3	35	13 (0)	37% (0%)	13 (0)	37% (0%)	5%

a Regenerated T0 plants (Table 5) that survived transplanting to soil were analysed for edits.

b Edits are defined as targeted mutagenesis and include both in‐frame and frameshift mutations.

c A knockout is defined as targeted mutagenesis that results in a frameshift mutation.

d See Table 5 for the number of embryos transformed per gRNA experiment.

## Discussion

Sorghum has long been considered one of the most recalcitrant crops for *Agrobacterium*‐mediated gene delivery, tissue culture, regeneration and genetic transformation. This situation has been significantly improved through efforts on tissue culture media optimization, as described by Wu *et al*. (2014) and through gene delivery system improvements as described in this report. The use of a ternary vector system containing the T‐DNA binary vector and the optimized pVIR accessory plasmid significantly improved the efficiency of sorghum transformation compared to the previously described CIV vectors. The ternary vector not only simplifies vector construction due to the smaller size of cloning vector carrying the T‐DNA but also significantly increases the gene delivery capability which ultimately improved transformation efficiency in both corn (Anand *et al*., 2017b) and sorghum. Together with surfactant application for the immature embryo infection, the ternary vector was able to achieve 29% transformation frequency with the *PMI*/mannose selection system (Table 2) and around 26% based on the *NPTII*/G418 selection system (Table 3) in inbred line Tx430. In addition, the ternary vector system allowed stable transformation of large size T‐DNAs containing four different proprietary genes (T‐DNA up to 18 kb) without adversely affecting transformation frequency and quality event frequency (Tables 1, 2, 3), thus making the ternary vector system even more attractive for genome engineering.

A new nonantibiotic and nonherbicide system was developed for sorghum transformation based on *PTXD* as the selectable gene. Despite somewhat lower transformation frequency (Table 4), *PTXD/*Phi selection system is nonetheless an attractive transformation system that can be further improved, for example through monocot codon optimization of the *PTXD* gene to enhance the positive selection efficiency.

To further explore the transformation potential mediated for different sorghum varieties, we further applied the ternary vector system to three African varieties, namely *Macia*,* Tegemeo* and *Malisor 84‐7*, and achieved efficient transformation frequencies as well (Table 2). The optimized ternary vector system allows us to introduce transgenes directly into these Africa varieties without trait introgression backcrossing, a time‐consuming process that typically takes many years to achieve and often results in yield drag (Mumm, 2013).

Furthermore, the ternary vector system containing the accessory plasmid pPHP71539 made it possible to generate targeted genome modification using CRISPR/Cas genome editing technology with a knockout efficiency ranging from 1% to 5% for the biallelic lethal gene, *Sb‐CENH3* (Table 6 and Table S2). The success of targeted genome modification in sorghum provides a powerful tool for studying sorghum genetics which will contribute to elucidating sorghum heat‐ and drought‐resistant mechanisms and improving its yield and grain quality in sorghum in the near future.

Overall, the broad applications of the ternary system with pPHP71539 as the accessory plasmid described in this study makes *Agrobacterium*‐mediated sorghum transformation become more practicable and can easily be implemented by most laboratories.

## Experimental procedures

### Constructs for transformation

The CIV research construct pPHP38332 carrying the *PMI* selectable marker gene and YFP reporter gene has been previously described by Wu *et al*. (2014) (Figure S1a). The pVIR accessory plasmid pPHP71539, described by Anand *et al*. (2017b), comprises a pVS1 ORI and gentamycin as the bacterial selectable marker. The binary plasmids pPHP45981 (Figure S1b), pPHP81561 (Figure S1c) and pPHP70444 (Figure S1d) contain the repABC ORI, the spectinomycin bacterial selectable marker, the YFP reporter gene and *PMI*,* NPTII* or *PTXD,* respectively, as plant selectable marker genes, respectively. pPHP78152 and pPHP78233 are production constructs derived from pPHP82637 (Figure S1e) with proprietary genes with varying size of T‐DNA (17 and 18 kb, respectively). The ternary design was assembled by first mobilizing the accessory plasmid pPHP71539 in the *Agrobacterium* auxotrophic strain LBA4404 Thy‐ and selected on media supplemented with gentamycin (25 mg/L). Subsequently, the binary constructs were electroporated into *Agrobacterium* strain LBA4404 Thy‐ containing the accessory plasmid and recombinant colonies were selected on media supplemented with both gentamycin and spectinomycin. All constructs were then subjected to next‐generation sequencing and sequence confirmed before conducting transformation experiments.

CRISPR/Cas gene editing was achieved using the LBA4404 Thy‐ *Agrobacterium* strain and the pPHP71539 pVIR accessory system described by Anand *et al*. (2017b). The spCas9 and sgRNA gene editing machinery and the *NPTII* selectable marker were expressed on a TDNA expressing binary vector (pPHP82151) (Figure S3). The N20 region labelled as the crRNA DNA‐hybridization region represents RNA sequence used to target genomic sequence in the located upstream of the DNA triplet ‘NGG’ sequence in the *Sb‐CENH3* gene that is recognized by the spCas9 enzyme.

Materials reported in this article may contain components subject to third‐party ownership (e.g. PTXD, moPAT, PMI). Transgenic and genome edited materials may be subject to governmental regulations. Availability of materials described in this article to academic investigators for noncommercial research purposes under an applicable material transfer agreement will be subject to proof of permission from any third‐party owners of all or parts of the material and to governmental regulation considerations. Obtaining the applicable permission from such third‐party owners will be the responsibility of the requestor. Transgenic materials reported in this article may only be made available if in full accordance with all applicable governmental regulations.

### Sorghum transformation and transgenic event quality analysis

Sorghum genotype Tx430 and African varieties (*Macia*,* Malisor 84‐7* and *Tegemeo*) grown in a greenhouse were used in this study. *Malisor 84‐7* (PI 656048) and *Macia* (PI 565121) were requested from USDA GRIN. *Tegemeo* is a public line obtained from Mycogen Seeds. Immature embryo explants isolated from those sorghum plants were transformed with *Agrobacterium* auxotrophic strain LAB4404 Thy‐ carrying above vectors to generate transgenic sorghum plants. The same *Agrobacterium* transformation methods were performed as previously described by Wu *et al*. (2014), with the exception that the resting step was skipped for *PTXD*/Phi selection system and KH_2_PO_4_ in the callus development medium was replaced with 300 mg/L Phi.

The integrated copy number of the T‐DNA and the vector backbone in these transgenic plants were determined by a series of qPCR analyses (Wu *et al*., 2014; Zhi *et al*., 2015). The transgenic plants carrying a single copy of the intact T‐DNA integrations without vector backbone were defined as ‘quality events’.

### Amplicon deep sequence

CRISPR/Cas edits were characterized by amplicon sequencing from DNA extracted from a single fresh leaf punch from each plant as per manufacturer's recommendations via the sbeadex™ tissue extraction system (LGC Limited, UK). DNA was normalized to 10 ng/μL, and twenty cycle target region PCR was performed on 50 ng of genomic DNA with Phusion™ Flash 2× Master Mix (Thermo Scientific, Waltham, MA) as per the manufacturer's recommendations. Five microlitres of primary PCR product were transferred to the twenty cycles secondary amplification containing primers to attach individual sample indexes and sequencing components, again with Phusion™ Flash 2× Master Mix. Sequencing was performed on Illumina MiSeq^®^, paired‐end 150 cycles per read according to Illumina standard operating procedure. Sequence reads were aligned to the wild‐type reference sequence via Bowtie2. The primers used to amplify *Sb‐CENH3* genomic loci are sites in Table S1. Edited sequences are reported in Table S2.

## Author contributions

P.C., A.A., E.W., J.S., M.S., A.S., M.M., D.L., S.L., Z.Z., M.A. and T.J. designed research; W.Z. conducted sorghum transformation; P.C., A.A., E.W., J.S., M.S., A.S and. G.Z. analysed data; P.C., A.A., E.W., J.S. and T.J. wrote the manuscript.

## Conflict of interest

The authors have no conflict of interest to declare.

## Supporting information


**Figure S1** Schematic representation of the molecular components of constructs used in this study (see Experimental Procedures for details).
**Figure S2** Tx430 *Sb‐CENH3* genomic structure. The 5′ UTR is highlighted in green. The exons are highlighted in yellow and the 3′ UTR is highlighted in grey. Introns are represented by lowercase letters.
**Figure S3** Schematic representation of the molecular components used for gene editing in this study.
**Table S1** Primers for CRISPR/Cas target sites.
**Table S2** Sequences changes from Cas9 edited plants.
