# Supplementary material for: Developing a flexible, high‐efficiency Agrobacterium‐mediated sorghum transformation system with broad application
Source: Plant Biotechnol J. 2018 Feb 6;16(7):1388–95. doi: 10.1111/pbi.12879 (PMC5999184; doi:10.1111/pbi.12879)
Supplement: Supplementary file 1 — Figure S1 Schematic representation of the molecular components of constructs used in this study (see Experimental Procedures for details). Figure S2 Tx430 Sb‐CENH3 genomic structure. The 5′ UTR is highlighted in green. The exons are highlighted in yellow and the 3′ UTR is highlighted in grey. Introns are represented by lowercase letters. Figure S3 Schematic representation of the molecular components used for gene editing in this study. Table S1 Primers for CRISPR/Cas target sites. Table S2 Sequences changes from Cas9 edited plants. [file PBI-16-1388-s001.docx]

**Supporting information**

**Table S1** Primers for CRISPR/Cas target sites

| Gene Target | DNA Target Sequence | Forward Primer | Reverse Primer |
| --- | --- | --- | --- |
| Sb-CENH3.1 | GAACCAAGCACCAGGCCGTGaGG | GCCCGAGAGTTCTGAATCG | GGAGCTTCTTCTTGGGCTTC |
| Sb-CENH3.2 | TGCAGGTGGGGCGAGTACGTcGG | GCTAAGCGCGTTCCTTTTT | GCAAAAGGAGGGCTAAAACC |
| Sb-CENH3.3 | TGCAGCGCAAGGAGGGCTTCaGG | CTGGAGAATAGGGCGCTACA | CCAGACCGATTAACAGAAAAAGA |

**Table S2** Sequences changes from Cas9 edited plants.

| Target Site | Plant # | Allele Change | **Δ** |
| --- | --- | --- | --- |
| Sb-CENH3.1 | 1 | -AAGCACCAGGCCGTGAGG | -18 bp |
|  | 1 | -C | -1 bp |
|  | 2 | -CCAGGCCG | -8 bp |
|  | 2 | -C;+TCGAGCGCGCAGGTTA | +15 bp |
|  | 3 | Wildtype |  |
|  | 3 | -CGT;+G | -2 bp |
|  | 4 | -GCTCGAACCAAGCACCAGGCCGT | -23 bp |
|  | 4 | Wildtype |  |
|  | 5 | Wildtype |  |
|  | 5 | +T | +1 bp |
|  | 6 | +C | +1 bp |
|  | 6 | Wildtype |  |
|  | 7 | Wildtype |  |
|  | 7 | +C | +1 bp |
|  | 8 | -GTG | -3 bp |
|  | 8 | Wildtype |  |
|  | 9 | -GGCCG | -5 bp |
|  | 9 | Wildtype |  |
|  | 10 | -AGCACCAGGCCG | -12 bp |
|  | 10 | +ATCACGCGCTGCCCCCTTTCTTCCTCGCGCTGCCCCCTT | +39 bp |
|  | 11 | -AAGCACCAGGCCGTG | -15 bp |
|  | 11 | -GGCCGT | -6 bp |
|  | 12 | -GCCG;+TGTC | 0 bp |
|  | 12 | +C | +1 bp |
| Sb-CENH3.2 | 1 | -CGT | -3 bp |
|  | 1 | -GCAGGTGGGGCGAGTACG;+TTTTTTCCT | -9 bp |
|  | 2 | -GGCGAGTACGTC | -12 bp |
|  | 2 | +T | +1 bp |
|  | 3 | -CG | -2 bp |
|  | 3 | Wildtype |  |
|  | 4 | -GGCGAGTACGTC | -12 bp |
|  | 4 | -ACGTCGG | -7 bp |
|  | 5 | -GGCGAGTACGTC | -12 bp |
|  | 6 | -GGCGAGTACGTC | -12 bp |
| Sb-CENH3.3 | 1 | Wildtype |  |
|  | 1 | -A | -1 bp |
|  | 2 | -CCTGAAG | -7 bp |
|  | 2 | Wildtype |  |
|  | 3 | Wildtype |  |
|  | 3 | +AA | +2 bp |
|  | 4 | Wildtype |  |
|  | 4 | -AG | -2 bp |
|  | 5 | -CCTGAAG | -7 bp |
|  | 5 | Wildtype |  |
|  | 6 | +T | +1 bp |
|  | 6 | Wildtype |  |
|  | 7 | +A | +1 bp |
|  | 7 | Wildtype |  |
|  | 8 | -gccctccttgcgctgcaagaggtcagttatgaa | -33 bp |
|  | 8 | Wildtype |  |
|  | 8 | +A | +1 bp |
|  | 8 | +G | +1 bp |
|  | 8 | +T | +1 bp |
|  | 9 | Wildtype |  |
|  | 9 | +A | +1 bp |
|  | 10 | Wildtype |  |
|  | 10 | +A | +1 bp |
|  | 11 | Wildtype |  |
|  | 11 | +A | +1 bp |
|  | 12 | Wildtype |  |
|  | 12 | +A | +1 bp |
|  | 13 | Wildtype |  |
|  | 13 | +A | +1 bp |

Frameshift alleles are highlighted in yellow.

(a) (b)

pPHP45981

SPC (VER2)

REP C (AR) (MOD1)

REP B (AR)

REP A (AR) (MOD1)

VIR G (ALT1)

ZS-YELLOW1 N1

PMI (PHI)

UBI1ZM INTRON1 (PHI)

UBI1ZM INTRON1 (PHI)

ATTB2

ATTB3

ATTB4

RB

MINI-ALLSTOPS3

MINI-ALLSTOPS2

MINI-ALLSTOPS

MINI-ALLSTOPS4

LB

UBI1ZM PRO

UBI1ZM PRO

COLE1 ORI

PINII TERM

PINII TERM

UBI1ZM 5UTR (PHI)

UBI1ZM 5UTR (PHI)

pPHP38332

TET

TET A

TRF A

CTL

VIR C1

VIR C2

VIR G

VIR B

PMI (SYN)

MO-PAT::ZS-YELLOW1-N1

SPC

UBI1ZM INTRON1 (PHI)

UBI1ZM INTRON1

RB

LB

UBI1ZM PRO

UBI1ZM PRO

ORI V

COLE1 ORI

COLE1 ORI

PINII TERM

PINII TERM

UBI1ZM 5UTR

UBI1ZM 5UTR

COS

COS

pPHP70444

ZS-YELLOW1 N1

PTX D (MOD3)

MO-PAT (MOD1)

REP A (AR) (ALT1)

REP B (AR)

REP C (AR) (MOD1)

SPC (VER2)

UBI1ZM INTRON1 (PHI)

UBI1ZM INTRON1 (PHI)

OS-ACTIN INTRON1 (MOD1)

RB

MINI-ALLSTOPS3

PSA2

ATTB4

ATTB2

FRT1

ALL STOPS2

ATTB3

PSB1

MINI-ALLSTOPS4

LB

UBI1ZM PRO

UBI1ZM PRO

OS-ACTIN PRO

PUC ORI

SB-GKAF TERM (MOD1)

PINII TERM

CZ19B1 TERM

CAMV35S TERM

UBI1ZM 5UTR (PHI)

UBI1ZM 5UTR (PHI)

pPHP81561

ZS-YELLOW1 N1

NPTII (MO1)

REP A (AR) (ALT1)

REP B (AR)

REP C (AR) (MOD1)

SPC (VER2)

UBI1ZM INTRON1 (PHI)

UBI1ZM INTRON1 (PHI)

RB

BUFFER1

MINI-ALLSTOPS3

PSA2

ATTB4

ATTB3

PSB1

MINI-ALLSTOPS4

LB

UBI1ZM PRO

UBI1ZM PRO

PUC ORI

PINII TERM

SB-ACTIN TERM (1.1KB)

PINII TERM

UBI1ZM 5UTR (PHI)

UBI1ZM 5UTR (PHI)

(c) (d)

pPHP82637

CCDB

CAM

PMI (SYN)

REP A (AR) (ALT1)

REP B (AR)

REP C (AR) (MOD1)

SPC (VER2)

UBI1ZM INTRON1 (PHI)

RB

BUFFER1

MINI-ALLSTOPS3

PSA2

ATTR4

CCDB ENH

ATTR3

MINI-ALLSTOPS

ALL STOPS2

PSB1

MINI-ALLSTOPS4

LB

UBI1ZM PRO

PUC ORI

FL2 TERM (ALT1)

PINII TERM

CZ19B1 TERM

UBI1ZM 5UTR (PHI)

LOXP

LOXP

(e)

**Figure S1** Schematic representation of the molecular components of constructs used in this study.

TGGTACACGGACAAAGGTTAGCGGTCACCGCGAATCGTGAATACTTGTGACTACGGGGTGCTAATTATAAAAACGCCGCACATCCTTTCGTTTCGCCATTTCACCCCCCTTCCCTTCCCGTAGAGAG_AAAAAAACCCACCGTCGACCCGCTCGGCCGCCCGAGAGTTCTGAATCGAAACCGTCGCCCGCGACCGCGAGAGCAGCGCGGGGCGCCCACCGTGATGGCTCGAACCAAGCACCAGGCCGTGAGGAAGCTGCCGCAGAAGCCCAAGAAGAAGCTCCAGTTCGAGCGCGCAGgtaagcccgcgtccccgcgctgaacccccctccgcctcgcgagcagacgctgccgctgctctccgtcgcccctggtgctaagcgcgttcctttttttttccttcttttgcagGTGGGGCGAGTACGTCGGCGACCCCGgtgagtgcgtgcgtgcgggaattggttttagccctccttttgcggtttcgccttttgttgggctggtctcacttgcttgcaatctgtttgatggaatgcagGAGAGGAGGAATGCTGGGACCGGGGGAGGAGCCGCGGCTCGCGgtgaggatctctttgtcgttgctgggtttgggaatttccggcgcgaaattatgtggatttctaggtttatctgccgtctttcttcttgtcttctcttttggctctggggtgagaagttagggtggttgggcggacatggtgcgttatttcgccgcatcgtttggtttggtgctttctcatccttttaattccaacatgccttgtaaaaattgcacaggatttgttttttcatgcatgtctcagtgttgctaatttgcttttccggttcagttggtagaattcaatttcttggcgcaatatgcatcttcttttgttgcaacatgagggcgaatgtgccagttccatatgggcgtcgcggttttgaagttactaccttgcttgctcttcgtattataggcgtcattcacaatagtatgttttcttggagatgcagTTGCACGGGGGCGTGTGGAGAAGAAGCATCGCTGGCGGGCAGGGACTGTAGCGCTGCGGGAGATCAGGAAGTACCAGAAGTCCACTGAGCCGCTCATCCCCTTTGCGCCCTTCGTACGTGTGgtgggtgcatcttgtaccaattgttgtccactccatagaatgggtttgttctgcagtctgtctgatggaaagttattcttctgagaaaaaatgcagGTCAAAGAGTTAACTGCATTCATAACAGACTGGAGAATAGGGCGCTACACCCCTGAAGCCCTCCTTGCGCTGCAAGAGgtcagttatgaaacatgtcttgtgtatcagttaagatcatcttctatagacataattgttatcatgaagtctttttctgttaatcggtctggtactacttaataatcaggatttcagattgctgcctttcctagtggtgtagtcaaaagggaatttaagtgctgttaggtactgtttgttttggtgttttgaaccctgccgcgatcggttgttgttattccatgtttgtttctgtggcagcggacgttcacggtgagatgggatacgggcgtgtgaaacatagttacggtccatcttcatggcttatccatttacgctgctcgtccgctcacttgttatgtgcggcaaccaaacttttgttactagtgtaactggtagcgttgcaaatctttccatttgcgttaccactccctatgggagccaaacagcaccttagtgtagattccatttgtattacttgagctagcttccttgctattggtgcctcgattgtactgttatgatcgaagtgctgaaaactttgtcgcctgcatagcatgattagagaacttgagtttacatttattcaataccttaagactgcatttcgtatagataaattatttttcctaattgttctggttaactgttttaggtttccatatttttgtatgtgtatcatttaaattattgtgttgtttttcctccctgtctacagGCAGCAGAATTCCACTTGATAGAACTGTTTGAAGTGGCGAATCTGTGTGCCATCCATGCCAAGCGCGTAACAGTCAgtaagttatcactgaatgaactccttttcctctgtactattacgcctaatggagatgtgtgatgcatttttggttacacgattctttagtgattctgcttcagttggatatgataaatctagatgttatttaaagtggcaaattgcttacgagtggaaatagtaatgttcaaatagtgaaaagtgcaattaaacttttaataggccattatatggtttgattgtcaacaaatgcatcaagaaatagtaaatattataacagttatggcttagagagtggacaaaaaatcggtaatggtgagctttgtataaacactaaaactggctgagaaatctgataactcaaggatctataggaaatgtattatcctaaatgttttccttcctgctgcagTGCAAAAGGACATACAACTTGCAAGGCGTATCGGAGGAAGGCGTTGGTCGTGATATCCATTCTGATTCTGATTACCTTGTTCGGGTGGAATTTGTTTAGAGGAGTTAGACATTAGTCTTGTTGAATGCTGTGCATGGTTCCTAATCTGTTTCACAGTTAGTGGGCTCTTCTGGGATGATCTGTTAACACCTGTGGAGTATGTTATGTAGGAAACACCTGAACTGAACAACCCAAAGTTGTTTTGGTTGCTCTTCAACCATTTGTTTGCTTCAGAGATCGATTCTAAACTGCATGCTAATTAGTCTATGGTTGAACAAAAATTATCAAATATAAATGAAAGTGATATAGTAGCA

**Figure S2** Tx430 *CENH3* genomic structure.

pPHP82151

SPC (VER2)

SV40 NLS

VIRD2 NLS

NPTII

AM-CYAN1

REP A (AR) (ALT1)

REP B (AR)

REP C (AR) (MOD1)

CAMV35S ENH

UBI1ZM INTRON1 (PHI)

ST-LS1 INTRON2

ST-LS1 INTRON2-V2

UBI1ZM INTRON1 (PHI)

RB

MINI-ALLSTOPS3

PSA2

ATTB4

ATTB1

GUIDE RNA

ATTB3

ATTB1

ATTB2

MINI-ALLSTOPS

ALL STOPS2

PSB1

MINI-ALLSTOPS

MINI-ALLSTOPS4

LB

UBI1ZM PRO

ZM-U6 POLIII CHR8 PRO

RAB17 PRO

UBI1ZM PRO

LTP2 PRO

PUC ORI

PINII TERM

ZM-U6 POLIII CHR8 TERM

PINII TERM-V3

FL2 TERM (ALT1)

PINII TERM

CZ19B1 TERM

CAMV35S TERM

UBI1ZM 5UTR (PHI)

RAB17 5UTR

UBI1ZM 5UTR (PHI)

SB-CENH3-CR1

CAS9 EXON1 (SP) (MO)

CAS9 EXON2 (SP) (MO)

MO-CRE EXON1

MO-CRE EXON2

LOXP

LOXP

**Figure S3** Schematic representation of the molecular components used for gene editing in this study.
